# On the Exclusion of Atmospheric Air in the Treatment of Certain Local Diseases

**Published:** 1844-10

**Authors:** Marshall Hall


*On the exclusion of Atmospheric Air in the treatment of certain local diseases.* By Marshall Hall, M.D.—Some years ago I attended a fatal case of peritonitis. On a post-mortem examination I was struck with the florid-red appearance of those parts of the intestines which were contiguous and adherent to the abdominal parietes, and the perfectly pale condition of those other parts of the intestinal canal which were contiguous and adherent to each other. Both surfaces were equally covered with a layer of rather opaque and moderately consistent coagulable lymph. I could only account for the difference in the appearance of these two portions of the same intestine by supposing that one was affected by the absorption of oxygen from the atmospheric air, whilst from the other this gas was excluded.

It is usual in the Parisian hospitals to trust the treatment of pleuritis greatly to the application of cataplasms. I confess that when I first heard of this mode of treatment I thought it trifling. I have since considered that these cataplasms may entirely exclude the influence of the atmospheric air, and thus prove of real efficacy. But whatever may be the *rationale*, the fact remains as I have stated it, and where the treatment of pleuritis consists greatly in the application of mere cataplasms, a post-mortem in this disease is scarcely or not to be obtained, so generally do the patients recover.

I have now to add a fact from my own personal experience. I have recently seen the most satisfactory result, both in pleuritis and peritonitis, from the assiduous application of cataplasms of powdered linseed.

It is probably by the exclusion of the atmospheric air that other remedies for inflammatory diseases act; the various plasters, the nitrate of silver, even blisters, have this effect. I do not, however, mean to insinuate that they have no other. Cataplasms may further act by their warmth and moisture. The nitrate of silver possesses some extraordinary power over the actions which constitute or coincide with inflammation. But, certainly, mere adhesive plasters have an efficacy in cases of chronic chest affection, in lumbago, sciatica and other forms of rheumatism, in neuralgia and even of scirrhus, which cannot be easily explained.

One of my patients, a martyr to extensive sciatica, was desired to envelop the limb in adhesive plaster. He was a joiner, and an ingenious man. He prepared the common stocking material with glue, dissolved in the proportion of one ounce to two pints of water, and had it spread over, when dry, with galbanum plaster, and if this exuded it was dusted with flour. By the steady application of this plaster his severe rheumatism was cured.

I was once informed by a celebrated physician that he had prescribed adhesive plaster to be applied over a scirrhous tumor of the mamma. It remained adherent for years, and the disease remained stationary. The plaster then separated, and from that period the disease pursued its devastating progress.

Certain modes of the treatment of burns consist in excluding the influence of atmospheric air.

Some affections of the face are remedied by applying a layer of gelatine. Isinglass is dissolved in water, and the solution is applied with a camel's-hair pencil, and allowed to dry. I have just witnessed some very remarkable effects of this mode of treatment, which I will communicate hereafter.—*Med. News—from the Lancet.*

